# Microfluidic approaches for liquid biopsy in glioblastoma: Insights into diagnostic and follow‐up strategies

**DOI:** 10.1002/btm2.70032

**Published:** 2025-05-22

**Authors:** Clara Bayona, Teodora Ranđelović, Claudia Olaizola‐Rodrigo, Ignacio Ochoa

**Affiliations:** ^1^ Tissue Microenvironment (TME) Lab Instituto de Investigación Sanitaria de Aragón (IIS Aragón), Instituto de Investigación en Ingeniería de Aragón (I3A), Universidad de Zaragoza Zaragoza Spain; ^2^ Centro de Investigación Biomédica en Red de Bioingeniería, Biomateriales y Nanomedicina, Instituto de Salud Carlos III Zaragoza Spain; ^3^ Beonchip S.L. Zaragoza Spain

**Keywords:** diagnosis, glioblastoma, liquid biopsy, microfluidics, non‐invasive monitoring, tumor biomarkers

## Abstract

Glioblastoma (GBM) is a highly malignant brain tumor with a poor survival prognosis of 12–15 months despite current therapeutic strategies. Diagnosing GBM is challenging, often requiring invasive techniques such as tissue biopsy and imaging methods that can provide inconclusive results. In this regard, liquid biopsy represents a promising alternative, providing tumor‐derived information from less invasive sources such as blood or cerebrospinal fluid. However, the typically low concentrations of these biomarkers pose challenges for traditional detection techniques, limiting their sensitivity and specificity. Recent advances in microfluidics offer a potential solution by enhancing the isolation and detection of tumor‐derived cells and molecules, thus improving their detectability. This review discusses the latest progress in microfluidic‐based liquid biopsy systems for glioblastoma, laying the basis for future diagnostic practices that are less invasive and more accurate. As these technologies evolve, they hold the potential to transform GBM diagnosis and monitoring, ultimately improving patient outcomes.

**Abbreviations**
APNG: alkylourine‐DNA‐N‐glycosylase
BBB: blood–brain barrier
cfNA: cell‐free nucleic scids
CTCs: circulating tumor cells
ctDNA: circulating tumor DNA
ctRNA: circulating tumor RNA
CNS: central nervous system
CSF: cerebrospinal fluid
EVs: extracellular vesicles
FDA: Food and Drug Administration
SPME‐GC–MS: solid phase microextraction gas chromatography mass spectrometry
GBM: glioblastoma
iMER: immuno‐magnetic exosome RNA
lnRNA: long non‐coding RNA
MGMT: O6‐methylguanine DNA methyltransferase
miRNA: microRNA
MRI: magnetic resonance imaging
MSP: methylation‐specific PCR
PDMS: polydimethylsiloxane
PET: positron emission tomography
POC: point‐of‐care
PYR: pyrosequencing
RANO: response assessment in neuro‐oncology
snRNA: mall non‐coding RNA
TEPs: tumor‐educated platelets
TR: tract recurrence
TME: tumor microenvironment
VOCs: volatile organic compounds
WHO: World Health Organization

**Translational Impact Statement**

This review highlights how integrating microfluidic technologies with liquid biopsy enables high precision biomarker detection and non‐invasive monitoring of glioblastoma, addressing critical challenges in its diagnosis and follow‐up. By enhancing the isolation and detection of low‐abundance tumor‐derived biomarkers in body fluids, these technologies hold significant potential to improve non‐invasive early diagnosis, guide therapeutic decisions, and transform patient management in glioblastoma and other inaccessible cancers. This work bridges bioengineering innovations with clinical applications, offering an impactful perspective for more precise and accessible diagnostic tools in oncology.

## 1 INTRODUCTION

Glioblastoma (GBM, WHO grade IV astrocytoma) is the most frequent, malignant, and aggressive primary brain tumor in adults, accounting for nearly 50% of malignant brain tumors across all age groups.^1^, ^2^, ^3^ According to the latest 2021 World Health Organization (WHO) classification, GBM is currently defined as a diffuse astrocytic glioma with no mutation in IDH genes nor histone H3 genes.^4^, ^5^ It is characterized by microvascular proliferation, necrosis, and specific molecular features, including TERT promoter mutation, EGFR gene amplification, and+7/−10 cytogenic signature.^4^ The tumor can arise de novo as a primary GBM or progress from lower‐grade astrocytomas as a secondary GBM, with distinct molecular profiles: primary GBMs frequently exhibit EGFR amplification and PTEN loss, whereas secondary GBMs are associated with TP53, ATRX, and PDGFRA mutations.^6^, ^7^ Other alterations, such as PIK3CA mutations linked to earlier recurrence and shorter survival,^8^ and MGMT promoter methylation status are also a widely accepted biomarkers in glioblastoma.^9^

Despite the advances in the current treatment—including surgery, concomitant radio‐chemotherapy, and adjuvant chemotherapy (Stupp protocol)^4^—patient prognosis remains very poor, with little progress over the past four decades in terms of prevention, early detection, and treatment.^3^ The median survival, even with Stupp treatment, is between 10 and 15 months, with most patients experiencing tumor relapse within 1 year of diagnosis. Moreover, the 5‐year relative survival rate has only increased from 4% to 7% during the past 40 years, and this drops to 2% in patients aged 65 years or older.^3^, ^10^

Early detection of a disease such as cancer is crucial, as it could lead to significantly improved patient outcomes. However, in the case of GBM, diagnosis typically occurs at an advanced stage due to the non‐specific nature of early symptoms, which overlap with those of more common, benign conditions. Clinical presentation varies depending on tumor size and location, with frequent symptoms including headaches, nausea (often related to increased intracranial pressure), fatigue, weakness, cognitive impairment, ataxia, and seizures. Given the vague nature of these symptoms and the limitations of current diagnostic tools, mainly due to tumor inaccessibility and the risks associated with invasive procedures, over half of patients are diagnosed only after emergency hospitalization when the tumor has reached an advanced stage.^10^, ^11^

This has led to a growing interest in alternative diagnostic and monitoring methods, such as liquid biopsy, which offers the advantage of being less invasive and potentially useful for repeated monitoring of disease progression. However, the low concentration of GBM biomarkers in circulation presents significant technical challenges for traditional detection methods. In recent years, microfluidic technology has emerged as a promising approach to address these limitations.

Microfluidic devices enable the precise manipulation of fluids at the microscale, offering superior sensitivity and specificity for the isolation, enrichment, and analysis of tumor biomarkers. These systems enhance the detection of circulating biomarkers from minimal biofluid samples, significantly improving diagnostic capabilities compared to conventional techniques. By reducing the sample volume required, increasing the efficiency of biomarker capture, and allowing real‐time analysis, microfluidic platforms facilitate early GBM detection and enable more frequent and accurate monitoring of disease progression.

In this review, we explore the latest advances in microfluidic‐based liquid biopsy technologies for glioblastoma, focusing on the main types of biomarkers—circulating tumor cells (CTCs), extracellular vesicles (EVs), circulating free nucleic acids (cfNAs), tumor‐educated platelets (TEPs) and volatile organic compounds (VOCs)—and the microfluidic devices developed for the detection of each, along with the specific application. By providing an overview of current challenges and future perspectives, we aim to highlight the role of these emerging technologies in improving early diagnosis, personalizing treatment strategies, and ultimately enhancing patient outcomes in glioblastoma.

## 2 CURRENT METHODS OF DIAGNOSIS IN GLIOBLASTOMA

### 2.1 Diagnosis methods: neuroimaging and tissue biopsy

Conventional approaches in diagnosis, subtyping, and monitoring brain cancers rely on advanced imaging and histopathological techniques. In imaging, there are several widely used methods, such as computer tomography, magnetic resonance imaging (MRI) and positron emission tomography (PET). Due to the high resolution and sensitivity compared to others, MRI is currently the most used.^12^ MRI includes T2‐weighted, T2‐weighted fluid‐attenuated inversion recovery sequences, and 3D T1‐weighted sequences before and after the application of a gadolinium‐based contrast agent.^13^ The integration of traditional and advanced MRI techniques with machine learning—an approach known as radiomics—is also emerging as a method to incorporate quantitative analysis into imaging interpretation. Radiomics encompasses features such as the location of recurrence and the volume of contrast enhancement, providing additional prognostic and diagnostic insights.^14^

PET, a nuclear medicine technique, is gaining relevance for GBM diagnosis and monitoring. It primarily employs two categories of radiotracers: glucose metabolism tracers (e.g., ^18^F‐FDG) and amino acid transport tracers (e.g., ^11^C‐MET, ^18^F‐FET, and ^18^F‐FDOPA). Although ^18^F‐FDG was initially the most extensively studied, amino acid PET tracers have demonstrated superior sensitivity in detecting gliomas and differentiating recurrent tumors from treatment‐induced changes.^15^, ^16^

However, despite the advancements in imaging technologies, the reliability of imaging assessment is still considered insufficient, as the appearance of glioblastoma on imaging scans can vary considerably. Therefore, all guidelines recommend obtaining a histological sample before deciding on therapeutic options.^4^ MRI is typically followed by resection or biopsy of tumor tissue to confirm the diagnosis, grade, and characterization of the tumor.^17^

### 2.2 Monitoring and follow‐up

Apart from diagnosis, tumor follow‐up is performed in an equivalent way. Clinical examination and MRI are the fundamental methods in the assessment of disease status and response to treatment, following the established Response Assessment in Neuro‐Oncology (RANO) criteria. After initial treatment, most patients undergo MRI scans at intervals of 2–6 months. However, routine neuroimaging is not typically indicated unless new neurological symptoms arise, and longer intervals between scans are often recommended to minimize patient risk from repeated exposure.^4^ This approach presents a significant issue for GBM patients, as tumor recurrence occurs in approximately 90% of cases.^18^ The lack of more frequent follow‐up could result in the late detection of recurrences, leading to tumor progression and a poorer prognosis. Therefore, there is a pressing need for more frequent, non‐invasive monitoring methods to improve early detection of relapses and better management of the disease.

### 2.3 Limitations of current diagnosis and monitoring techniques

Despite being the European Association of Neuro‐Oncology standardized diagnostic protocol, both neuroimaging by MRI and surgical resection have several limitations and complications. As previously explained, conventional MRI is the technique of choice for tumor detection and follow‐up, as it allows information on structure and location in guided surgery. However, it is only able to detect the solid tumor when it has sufficient mass, and it is difficult to distinguish between high‐grade gliomas (such as GBM and oligodendroglioma) or other diseases such as infections, lymphoma, and metastasis of an extracranial primary tumor. While PET imaging significantly improves the differentiation of gliomas compared to MRI, it still faces limitations in distinguishing oligodendrogliomas from GBM, as both exhibit similarly high amino acid uptake, complicating their classification.^19^

One of the key challenges for neurosurgeons is differentiating true tumor progression from treatment‐related contrast enhancement changes, known as pseudoprogression.^20^ It occurs frequently after combined chemo‐irradiation with temozolomide (TMZ), the current standard of care for glioblastoma. Pseudoprogression may increase contrast enhancement, caused by alterations in the blood–brain barrier (BBB) or radiation necrosis. Pseudoprogression occurs in 20%–30% of GBM patients, usually within the first 12 weeks of treatment, and it is important to differentiate it from actual progression to avoid unnecessary surgery or treatment.^21^, ^22^ Conversely, anti‐angiogenic agents can reduce contrast enhancement on MRI without a true antitumor effect by altering the permeability of the tumor vasculature, giving an erroneous perception of tumor shrinkage. This phenomenon is known as pseudo‐response.^20^, ^23^ Recent trials with VEGF‐related agents, such as bevacizumab, have shown a rapid decrease in contrast enhancement with a high response rate, but with rather modest antitumor effects.^20^ Both phenomena emphasize the limitations of MRI as a measure of tumor activity, as cases where there is no tumor progression, but an impaired BBB must be taken into consideration.

Although multimodal approaches integrating MRI, PET, and radiomics hold promise in improving the distinction between true progression and pseudoprogression, current techniques still require further validation in large‐scale, multicentre clinical trials before they can be reliably implemented as robust diagnostic tools.^24^ As a result, despite its invasiveness, tissue confirmation remains the gold standard for GBM diagnosis.^14^ Moreover, while advances in multimodal imaging techniques have improved lesion characterization, they provide only a partial representation of tumor properties, underscoring the need for novel diagnostic strategies that can yield deeper biological insights into tumor progression and therapeutic response.

Following the MRI, a tissue biopsy is performed to characterize the tumor and provide a more complete diagnosis. In the case of the brain, performing a biopsy involves a high degree of risk for the patient due to the invasiveness of the procedure.^25^, ^26^ Complications occurring after frame‐based stereotactic brain biopsies are rare but have serious side effects. The high invasiveness can lead to effects such as brain swelling or hemorrhages, which severely alter brain functionality.^27^ Despite being described as a minimally invasive procedure with a low complication rate, death has been reported as the most severe complication following frame‐based stereotactic brain biopsy.^26^ Moreover, there is an emerging risk associated with the biopsy procedure itself, tract recurrence (TR), where tumor cells may spread along the biopsy tract, a risk that has been previously underappreciated in brain metastases cases. Recent studies suggest that up to 50% of patients may develop TR after biopsy, highlighting the importance of careful radiographic monitoring and consideration of including the biopsy tract in adjuvant radiation therapy plans to manage this risk.^28^ On the other hand, the inaccessibility of some brain tumors and the small number of fragments that can be extracted make it difficult to obtain tissue samples that fully capture the great intratumoral heterogeneity.

In relation to the above‐mentioned, there is an urgent need in the clinic to identify less invasive detection methods for diagnosis and prognosis, and that in turn, can monitor tumor progression and response to therapy. Current techniques are not procedures that can be performed repeatedly over time to assess tumor dynamics and its molecular profile in real time, but only offer a static view of a tumor, which is known to undergo constant morphological and molecular changes.^21^

## 3 LIQUID BIOPSY IN GLIOBLASTOMA

In this context, liquid biopsy is increasingly being recognized as a potentially valuable tool for the identification and characterization of cancer biomarkers.^27^ It has demonstrated many favorable characteristics in this task, especially due to its minimally invasive and easy sampling nature.^12^ This diagnostic technique involves the analysis of biological fluids to identify and isolate molecules released by tumors into the general blood circulation and other bodily fluids, such as cerebrospinal fluid (CSF), saliva, and urine. While blood is the most commonly used in glioblastoma, CSF is also employed for biomarker detection.^29^, ^30^ CSF circulates in the brain and spinal cord and is therefore in close contact with the central nervous system. However, CSF collection requires an invasive lumbar puncture procedure.^21^ Among the most currently studied molecules are cfNAs (that includes circulating tumor DNA (ctDNA) and circulating cell‐free tumor RNA (ctRNA)), CTCs, EVs, VOCs, and TEPs.^27^, ^31^, ^32^ These molecules must be tumor‐specific and present in adequate concentrations for detection.

In addition to being less invasive than tissue biopsies, liquid biopsies have the potential to determine the genetic profile of cancer patients. For brain tumors, circulating biomarkers mean extracting useful information using a minimally invasive method. It has been reported that some of these molecules can cross the BBB and can be detected in the blood of GBM patients, even when their permeability is not impaired.^33^ These molecules can bypass tumor tissue and thus can provide a real sample of the tumor. In fact, a significant correlation has already been seen between the genetic profiles obtained from ctDNA by liquid biopsy and the corresponding tumor.^27^ Therefore, this method could be of great clinical utility, especially in cases where surgery is contraindicated, or biopsy results are inconclusive (approximately 25% of patients).^2^ Moreover, in cases of recurrence, which account for a remarkably high percentage, only 30% of patients are candidates for a second surgery. For this reason, circulating biomarkers could be used as a method of molecular diagnosis of recurrent tumors in inoperable patients, making it easier to identify the alterations that have led to recurrence and the possible treatments to be applied. The simplicity of the extraction method could be clinically relevant, as it enables early cancer detection through direct diagnosis and allows for the collection of serial samples that accurately reflect the tumor's evolution over time. This approach facilitates monitoring of potentially harmful changes in tumor aggressiveness, treatment response, and the risk of recurrence. Additionally, this procedure could allow the identification of disrupted signaling pathways, the molecular subtype classification, and also biomarker discovery.^12^

However, the technological limitations of detection have hindered the availability and knowledge of this technique until recent years.^34^ One key challenge is the low concentration of biomarkers in blood, making detection difficult. The isolation requires more complex and accurate procedures to obtain a sufficient quantity of cells, especially in early‐stage cancers.^35^ The localization of the tumor also affects the detectability of these biomarkers, with brain lesions, for instance, being difficult to assess through blood analysis.^36^ Microfluidic technologies offer a promising solution to these challenges by enabling the precise isolation and analysis of rare biomarkers, even in low concentrations. These technologies enhance sensitivity and reduce sample volume requirements, which is particularly beneficial for detecting biomarkers in difficult‐to‐reach tumors, such as those located in the brain.

## 4 MICROFLUIDICS FOR LIQUID BIOPSY IN GLIOBLASTOMA

Microfluidics involves the study and manipulation of fluids at the submillimetre scale, typically focusing on the precise control of small liquid volumes by exploiting specific physical properties of fluids.^37^, ^38^ It has proven to be a useful tool to improve diagnostic and biological research due to the increased sensitivity, reduced toxicity, biocompatibility, and enhanced drug delivery.^39^ By enabling miniaturization, microfluidics allows the integration of multiple analytical steps in a single platform, reducing the need for large sample volumes and reagents. This approach not only accelerates sample processing but also enhances detection sensitivity, making it particularly valuable for isolating and analyzing molecules or biomarkers that are present in minimal quantities or small sizes.^40^ Through these capabilities, microfluidics offers significant improvements in detecting low‐abundance biomarkers with high precision.

Microfluidic device fabrication methods can be broadly categorized into three main groups: photolithography‐based techniques, replication‐based techniques, and xurography. Of these, replication‐based approaches—including soft lithography, hot embossing, and injection molding—are the most extensively used in biomedical research due to their versatility and scalability.^41^, ^42^, ^43^ Most devices are composed of polydimethylsiloxane (PDMS), which offers good optical performance, biocompatibility, and flexibility, while enabling real‐time monitoring, high‐resolution imaging, and quantification of biological events.^44^, ^45^ However, it is a gas‐permeable material and retains non‐specific molecules. As an alternative, other materials have emerged, such as cyclic olefin polymer. This thermoplastic material, while retaining the benefits of PDMS, has a low gas permeability that allows for more precise control of the gases and is easier to handle on the production line.^46^, ^47^

Microfluidic technology is increasingly utilized in cancer research and can be used for personalized treatment and diagnosis. In this context, point‐of‐care (POC) biosensors emerged as innovative portable devices that enable rapid and precise disease detection outside the laboratory.^48^, ^49^ These biosensors, which include electrochemical and optical sensing platforms, have demonstrated significant potential in glioma diagnostics.^49^, ^50^, ^51^ Additionally, techniques such as PCR, RT–PCR, and high‐throughput single‐cell analysis have been extensively utilized in droplet‐based microfluidic platforms.^52^ These approaches have been applied to characterize gliomas based on their IDH1 mutational status^53^ and to distinguish glioblastoma tumor cells according to their proteolytic activity.^54^ Another major application is in the field of organ‐on‐chip, which uses microfluidics to closely mimic in vivo tumor conditions, providing an advanced preclinical tool to study personalized treatment for individual patients.^55^, ^56^, ^57^ Organ‐on‐chip devices have been developed to simulate glioblastoma microenvironment,^58^, ^59^ cell migration and invasion,^60^ metastasis,^61^ vascularisation and extravasation.^62^ Furthermore, they are increasingly being integrated into immuno‐oncology studies^63^, ^64^ and drug screening.^65^ Notably, the recent passage of the US Food and Drug Administration (FDA) Modernization Act 2.0, which removes the requirement for animal testing when in vitro alternatives like organ‐on‐chip demonstrate superior performance,^66^ has further fueled the adoption of these technologies.

Liquid biopsy is one of the most promising applications of microfluidics in GBM research. Although still an emerging field, different microfluidic devices are already available to optimize the detection and analysis of the main liquid markers (Table 1). These include CTCs, EVs, and VOCs. By reducing the need for invasive procedures for diagnosis and enhancing the accuracy of disease models, microfluidics addresses key challenges in GBM, allowing for minimally invasive biomarker analysis, improving sensitivity and reducing false positives, thus enabling more accurate and frequent diagnostic assessments.^67^ In the following sections, the main microfluidic approaches for each type of biomarker are discussed.

**TABLE 1:** Overview of microfluidic‐based liquid biopsy methods developed for the study of glioblastoma.

Type of biomarker	Clinical relevance in GBM	Microfluidic device	Description	Sensitivity/Specificity	Reference
CTC**s**	Tumor progression, monitoring, response to therapy	CTC‐iChip	Isolation of CTCs from whole blood using size‐based removal and inertial flow dynamics	Moderate (39%)/ High (80%)	Sullivan et al.^68^
		Anti‐EGFR aptamer device	Capture of GBM CTCs by anti‐EGFR aptamer functionalised substrate and optimisation of flow rate	High (70%)/ Very high (98%)	Wan et al.^69^
		Spiral microfluidic technology	Isolation of CTCs from whole blood and characterization with GFAP, vimentin and EGFR amplification	Moderate (65%)/ Very high (100%)	Müller Bark et al.^70^
		Parsortix™	Detection of CTCs clusters and exome sequencing	Moderate (53.8%)/ High (false‐positive threshold from healthy donors)	Krol et al.^71^
EV**s**	Tumor subtype classification, therapy resistance	Immunoaffinity‐based method	Isolation of GBM EVs using anti‐CD63 coated microchannels and detection of exosomal RNA	Moderate (42–94%)/ High (anti‐CD63 minimizes contamination)	Chen et al.^72^
		iMER	Comparison of exosomal mRNA levels of MGMT and APNG before and after TMZ treatment	Very high (93%)/ High (>95%)	Shao et al.^73^
		^EV^HB‐Chip	Isolation and detection of mutant EGFRvIII mRNA on EVs	Moderate (59%)/ High (94%)	Reátegui et al.^74^
		EZ‐READ	Profiling of circulating RNAs in EVs for blood‐based GBM characterization	High (LOD: 9 copies of miRNA)/ High (not specified)	Zhang et al.^75^
VOC**s**	Metabolic alterations, potential early biomarkers	Be‐Gradient device	Detection of in vitro TME VOCs in glioblastoma	High (not specified)/ Very high (exogenous VOCs removed)	Bayona et al.^76^

### 4.1 Circulating tumor cells in microfluidic models

CTCs are tumor cells that migrate from the solid tumor mass into the bloodstream. This is thought to be due to the induction of the epithelial‐mesenchymal transition in the tumoral cells, whereby the cells upregulate mesenchymal markers that give them invasive and migratory properties.^77^ CTCs contain genetic information of many types, as they harbor tumor DNA, RNA, and proteins, which allows useful information to be obtained to study tumor progression, invasion, and metastasis.^78^ However, in brain tumors, extracranial metastasis is not frequent, mostly due to the presence of the BBB and the short survival and infiltration of cells in a neutral environment.^79^ CTCs are found in very small amounts in the bloodstream (between 1 and 10 cells/10 mL of blood).^80^ In particular, the number of CTCs in GBM is estimated to be between 1 and 22 cells/2 million mononuclear cells.^81^ Therefore, the use of microfluidics for the isolation of CTCs may open the door to new diagnostic strategies for glioblastoma using affinity‐based and affinity‐free technologies,^82^ as these systems have also been shown to achieve higher purity in CTCs than other methods, such as density‐gradient centrifugation.^83^ Cell‐affinity chromatography uses a ligand with affinity for CTCs to selectively isolate these cells from the rest of the cells present in the blood.^84^ This technology was implemented in other tumors such as cervical cancer,^85^ to be adapted years later to the study of CTCs in different tumors such as melanoma, breast, colon, lung, and prostate cancer through the use of specific ligands.^78^, ^82^, ^86^ However, there is no certainty of total homogeneity in the expression of a particular marker, especially in a tumor as heterogeneous as GBM. Therefore, a negative selection could be an alternative to enrich the sample in CTCs from peripheral blood.^81^ These label‐free approaches are based on physical factors such as cell size and deformity,^87^ with the spiral microfluidic device being one of the options used in some head and neck,^88^ lung^89^ or breast cancer.^90^ On the other hand, there are commercial systems that provide a reliable system for the separation of CTCs. The CellSearch® system was the first FDA‐approved technology for the investigation of CTCs.^91^ It uses a ferrofluid consisting of a magnetic nanoparticle coated with anti‐EpCAM antibodies to capture cells of epithelial origin. However, in GBM, CTCs tend to have a mesenchymal phenotype, making this technology unsuitable for its study.^92^

**FIGURE 1:**
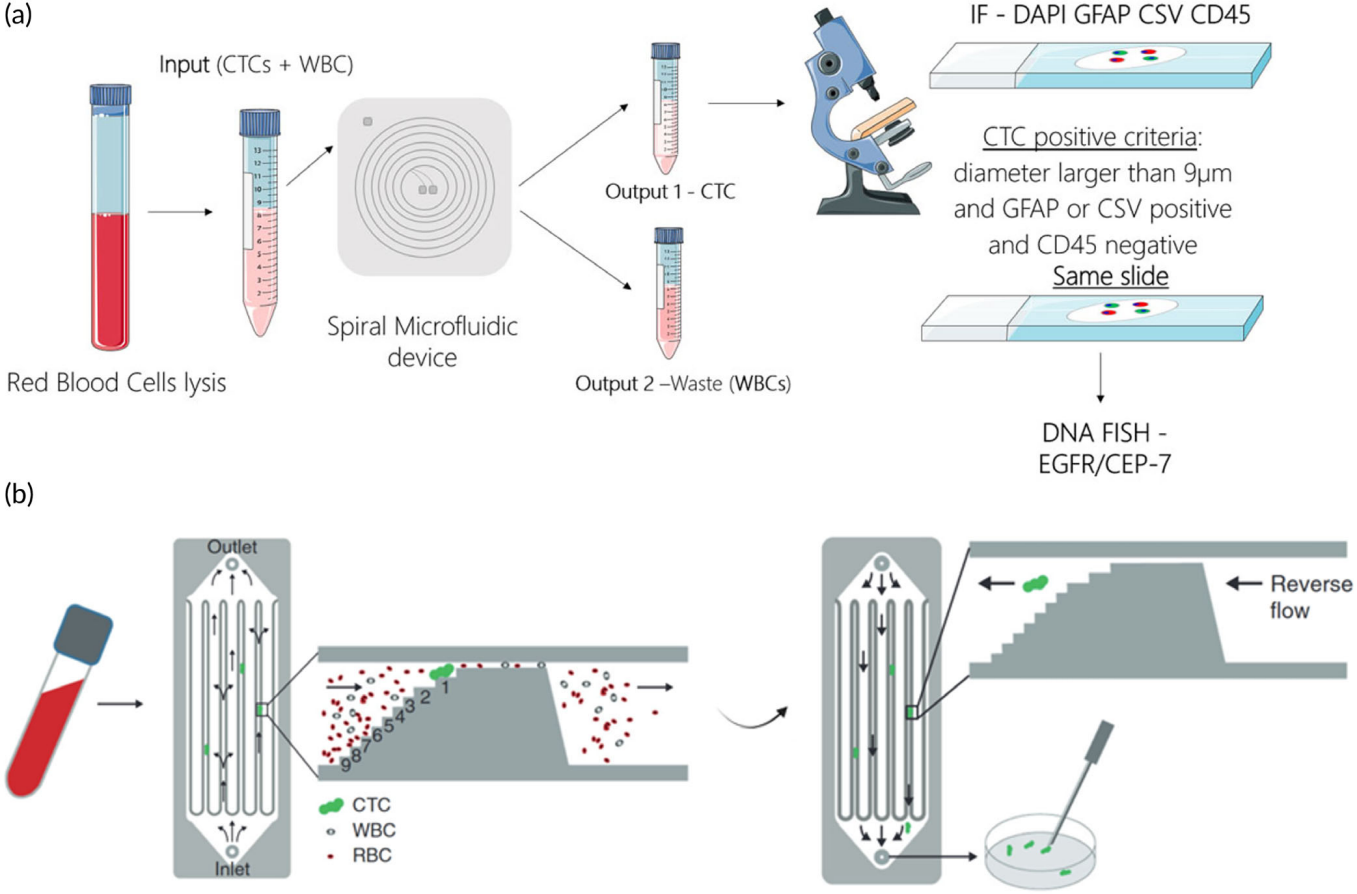
Microfluidic devices for the detection of CTCs in GBM. (a) Spiral microfluidic device developed by Müller Bark et al.^70^ to isolate and characterize GBM CTCs from the whole blood using inertial flow dynamics. This method enables detection of CTCs expressing GBM‐associated markers such as GFAP, vimentin, and EGFR amplification, which are relevant for tumor monitoring and characterization. (b) Parsortix™ microdevice used by Krol et al.^71^ for the identification of GBM CTCs clusters, facilitating tumor progression assessment and monitoring of therapy response. Reproduced under the terms and conditions of the Creative Commons Attribution (CC BY) license.

Despite being a tumor in which the presence of CTCs has been little studied in comparison to other tumors, there are studies that develop microfluidic techniques for the detection and identification of glioblastoma. In this context, Ozkumur et al. developed a microfluidic device called CTC‐iChip, which allowed the isolation of CTCs from whole blood.^93^, ^94^ Sullivan et al. applied this technology to GBM patients, obtaining CTCs in 13 of 33 patients. The study showed that CTCs were enriched in mesenchymal over neural differentiation markers compared with primary GBMs.^68^ Wan et al. developed a microfluidic device to study the capture of CTCs from GBM and the importance of fluid velocity for the isolation of these particles. Using this principle, they used anti‐EGFR aptamer functionalised substrate in the device to study the binding efficiency of CTCs. They found that a flow rate of 2 mm/s significantly facilitated the capture of CTCs.^69^ More recently, Müller Bark et al. reported the use of spiral microfluidic technology to isolate CTCs from the whole blood of newly diagnosed GBM patients before and after surgery (Figure 1a). This was followed by characterization with GFAP, vimentin, and EGFR amplification. They found CTCs in 13 of the 20 patients and demonstrated that patients without CTCs after surgery had a higher recurrence‐free survival.^70^ In addition, the Parsortix™ system described by Miller et al. was developed to capture cells according to their size and deformity, for subsequent analysis and characterization of the isolated cells.^95^ Krol et al. used this system to detect whether circulating cells could appear to form clusters and thus pass the BBB in 13 patients with progressing GBM (Figure 1b). They observed clusters varying from 2 to 23 cells at different stages of GBM progression. Exome sequencing of the CTC clusters revealed variations in 58 tumor‐associated genes such as *ATM*, *PMS2*, *POLE*, *APC*, *XPO1*, *TFRE*, *JAK2*, *ERBB4*, and *ALK*.^71^ As the articles in this field indicate, microfluidic systems for the detection of CTCs are a system that is progressing to offer more tumor‐specific techniques, despite the limitations that this implies for GBM. Commercial systems already exist for this purpose, and optimisation of sensitivity and isolation efficiency could help the implementation of these systems as an alternative diagnostic and monitoring method for hard‐to‐reach tumors such as GBM.

### 4.2 Extracellular vesicles in microfluidic models

EVs include all secreted or derived membrane vesicles shed by any type of cell, whose role is very varied; from intercellular communication in the microenvironment to proliferation, migration, drug resistance, and immunomodulation.^96^ They are a group of lipid‐bilayer bound nanoparticles ranging in size from 30 nm to 10 μm. This gives them a very important feature in the study of glioblastoma, since they are able to cross the BBB.^97^ These molecules can be extracted from plasma, serum, CSF, and even saliva. CFS collection involves an intrusive and difficult method,^98^ and saliva may show variability due to inconsistent collection and analysis methods.^99^ In blood, plasma is easier to obtain and can provide a high number of molecules to be studied. Exosomes and microvesicles are the two major classes of EVs that differ in their release mechanisms, size, and content (miRNA, mRNA, and DNA). Glioblastoma cells release EVs that participate in intercellular communication with the tumor microenvironment. These molecules have already shown promise as GBM biomarkers, providing insights into molecular characterization and subtyping for potential therapeutic and diagnostic applications.^100^

Different EV isolation techniques exist, each with its specific advantages and limitations. Ultracentrifugation is a common method that uses high‐speed spinning to separate EVs, with successive centrifugation at high forces eliminating dead cells and debris before pelleting the EVs. Density gradient ultracentrifugation, on the other hand, adds a density gradient to the centrifugation process, which allows for a more refined separation of EVs based on their buoyant density, resulting in higher purity.^101^ Other size‐based techniques, such as ultrafiltration and size exclusion chromatography, separate EVs based on their size, offering a gentler approach that preserves the integrity of the EVs. Additionally, commercial kits like ExoQuick™ and ExoQuick ULTRA provide precise and minimal sample methods for EV isolation.^98^, ^102^

Exosomes are molecules enriched in specific membrane markers such as CD63, CD81, and ALIX.^103^ Specifically, GBM‐derived exosomes differ from host exosomes by epidermal growth factor receptor (EGFR) amplification and other specific mutations such as EGFRvIII deletion.^104^ Studies using serum from GBM patients have revealed the presence of an inflammatory footprint by detecting EV biomarkers. Among the molecules found are VWF, FCGBP, C3, PROS1, and SERPINA1.^100^ In addition, upregulation of subtype‐specific biomarkers such as CD44, CD63, and CD81 has been found to be significant in GBM patients compared to controls. Besides, levels within GBM patients may vary, which could provide information on tumor subtypes or progression, bringing us closer to personalized treatment.^98^, ^100^

**FIGURE 2:**
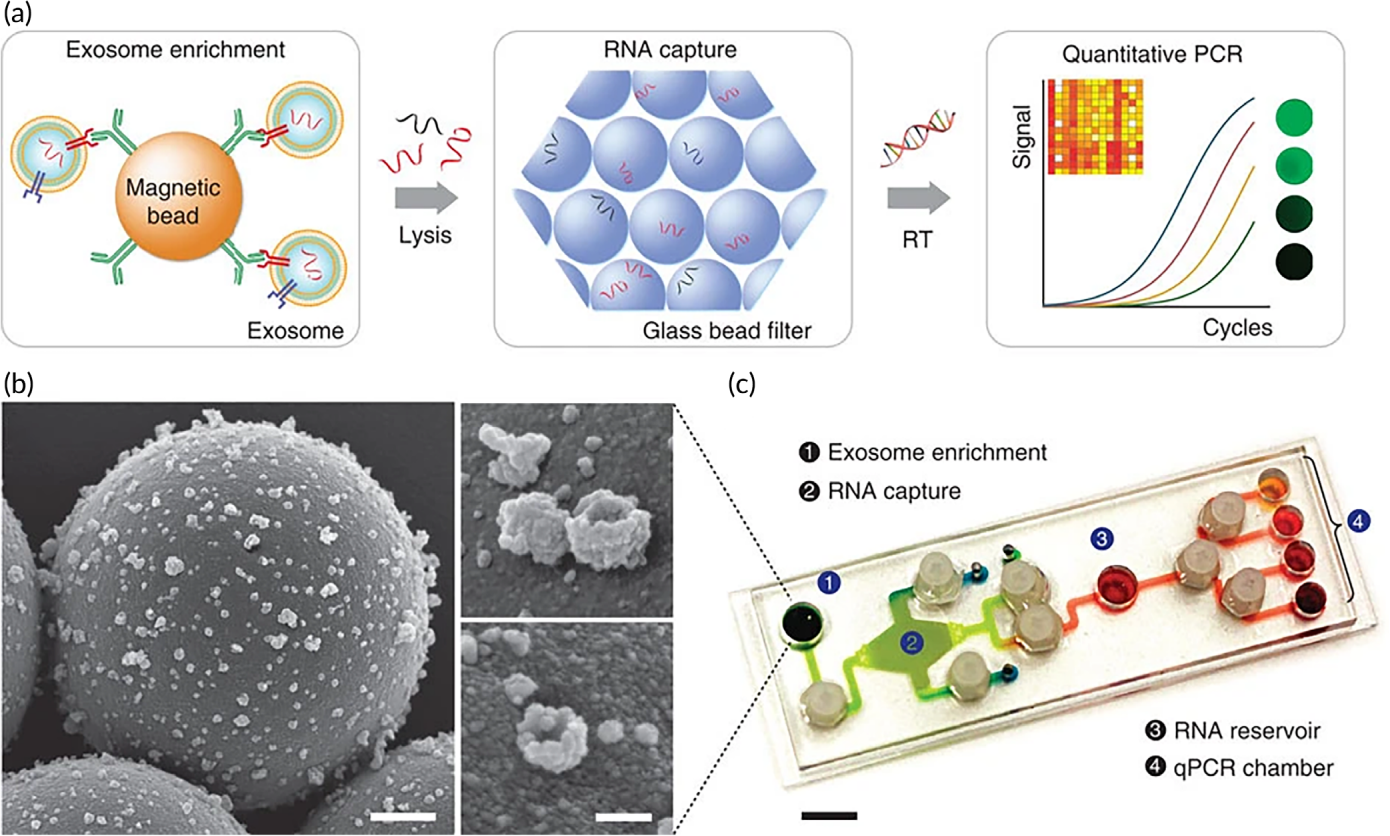
iMER platform developed by Shao et al.^73^ to characterize exosomal mRNA from blood samples. (a) The iMER platform streamlines exosome enrichment, RNA extraction, and real‐time analysis by capturing cancer exosomes on magnetic beads, isolating RNA via a glass bead filter, and enabling reverse transcription and quantification in a single device. (b) Scanning electron micrographs show antibody‐functionalized magnetic beads against EGFRvIII capturing tumor vesicles. (c) Image of the iMER device with (1) immunomagnetic capture site, (2) RNA extraction, (3) reverse transcription, and (4) qPCR chamber for multiplexed detection. Reproduced under the terms and conditions of the Creative Commons Attribution (CC BY) license.

The problem with current isolation methods is the need for an ultracentrifuge, in addition to the time‐consuming protocol, and simpler and faster methods would help in the characterization of GBM‐specific EVs. In this regard, microfluidic techniques can be useful to overcome some of the limitations mentioned.^105^ The immunoaffinity‐based method developed by Chen et al. for the isolation of EVs from GBM patients from small volumes of both blood samples and cultured cells is noteworthy.^72^ In the device, they used anti‐CD63‐coated microchannels to capture EVs, as they saw that it is overexpressed. Furthermore, they demonstrated that the quantity and quality of EVs captured were sufficient to detect exosomal RNA and assess tumor‐derived RNA. On the other hand, Shao et al. developed a system called immuno‐magnetic exosome RNA (iMER) analysis, which integrates immunomagnetic selection, TNA collections, and real‐time PCR in a single microfluidic device (Figure 2). In doing so, they compared the exosomal profiles of GBM samples after TMZ treatment and were able to detect exosomal mRNA levels of O6‐methylguanine DNA methyltransferase (MGMT) and alkylurine‐DNA‐N‐glycosylase (APNG) directly from blood samples, which may be potential predictive markers for the acquisition of TMZ resistance.^73^ Because of this, iMER could be used to examine diagnostic markers of GBM, in addition to allowing real‐time monitoring of drug efficacy. The same group developed other microfluidic platforms for the detection of exosomal proteins. Therefore, the use of these technologies prior to lysis and mRNA detection with iMER may be a viable method to extract as much information as possible from GBM samples.^106^, ^107^ In another study, a microfluidic device called the ^EV^HB‐Chip was used to isolate EVs from serum or plasma samples of GBM patients.^74^ They were able to detect and identify relatively rare EGFRvIII transcripts, as well as genes specific to GBM subtypes. Using the ^EV^HB‐Chip, they demonstrated 94% tumor EVs specificity and a detection level of 100 EVs per μL. Finally, the study of Zhang et al. developed an analytical platform to obtain direct and multiplexed profiles of circulating RNA in EVs for GBM characterization in blood. The technology, called ZIF‐8 enzyme complexes for regenerative and catalytic digital RNA detection or EZ‐READ, uses an RNA‐responsive transducer to regeneratively convert and catalytically enhance signals from rare RNA targets found in EVs in human blood. This technology allows the establishment of specific signatures for diagnosis and subtyping in the blood of GBM patients.^75^

### 4.3 Circulating tumor nucleic acids in microfluidic models

Within this category, several types of molecules can be found. cfDNA refers to DNA fragments of 150–200 base pairs that are shed into biological fluids primarily by apoptotic, necrotic, rapidly dividing cells and CTCs. cfDNA molecules have a short half‐life (<1.5 h), as they are quickly eliminated by phagocytosis. It is called ctDNA when the fragment belongs to a tumor cell.^108^ It accounts for 0.01%–10% of the overall cell‐free DNA in the blood, depending on the tumor's location in the blood, tumor activity, or the applied treatment.^109^ Typically, ctDNA levels in healthy individuals are low (around 10–15 ng/mL in plasma) compared to those with tumors. As the tumor expands, more cells release ctDNA.^110^ This molecule can be distinguished based on its size, increased fragmentation, and mutations that are not present in healthy cell DNA, making it a biomarker of disease.^111^ ctDNAs have been extensively studied in various cancer types and applications. In the case of colorectal cancer, they are widely used to evaluate the response and adaptation to treatment.^112^, ^113^, ^114^ In breast cancer, its existence has been investigated as a predictor for relapse,^115^, ^116^ response, and metastasis.^117^ Relevant findings on ctDNAs are also available for other types of cancer such as lung^118^ and prostate,^119^ among others. In the case of glioblastoma, certain limitations exist. A recent investigation discovered mutations in 55% of plasma samples from 222 GBM patients using NGS, which could offer viable options for identifying therapeutic alternatives based on genomic research by ctDNA.^120^ However, in most studies, the concentration of ctDNA extracted was lower in comparison to other tumors because of the presence of the BBB.^110^, ^121^ In contrast, CSF seems to be abundant in these substances, as displayed in Wang et al. research.^122^ They reveal that ctDNA can be identified through whole exome sequencing, enabling a wide‐ranging analysis of the GBM ecosystem to be achieved without the use of more intrusive methods.

Methylation patterns in cell‐free DNA have also emerged as a promising genomic feature for detecting the presence of cancer and determining its origin.^123^ Studies have shown that methylation profiling of cell‐free DNA released from CNS tumors in blood allows for the detection of tumor‐specific molecular markers.^124^ Balaña et al. demonstrated that the methylation profiles of MGMT, p16, DAPK, and RASSF1A gene promoters in serum‐derived DNA from GBM patients closely correspond to those found in matching tumor tissue, highlighting the potential of cfDNA profiling for non‐invasive tumor characterization.^125^ Liu et al. examined promoter hypermethylation in MGMT, p16INK4a, TIMP‐3, and THBS1, observing 100% specificity in the correlation between DNA hypermethylation in serum and CSF with tumor tissue in gliomas.^126^ Moreover, they found that methylation levels of MGMT, p16INK4a, and THBS1 in glioma serum were predictive of poorer overall survival, while hypermethylation of MGMT and THBS1 in CSF served as prognostic factors for progression‐free survival. Similarly, Lavon et al. investigated MGMT, p16INK4a, TIMP‐3, and THBS1 promoter hypermethylation in low‐ and high‐grade tumor serum and found that MGMT promoter methylation was strongly associated with tumor heterogeneity, aggressiveness, and disease evolution.^127^ Estival et al. evaluated MGMT methylation status and its concordance across paired whole blood and GBM tissue samples using methylation‐specific PCR (MSP) and pyrosequencing (PYR). Their results revealed a lower sensitivity in detecting methylation marks in cfDNA compared to tumor tissue (average sensitivity of 31.5%), though the specificity of the MSP assay in blood reached 96%, while the PYR method showed a specificity of 76% in plasma.^128^ To improve detection accuracy, Barault et al. applied the Methyl‐BEAMing assay to plasma cfDNA from GBM patients, demonstrating high reproducibility, specificity, and sensitivity for the quantitative assessment of MGMT methylation.^129^

Apart from DNA, this category also includes ctRNA, which comprises mRNAs, long non‐coding RNAs (lnRNAs), and small non‐coding RNAs (snRNAs). The latter group encompasses microRNAs (miRNAs).^108^ miRNAs are small molecules of 19–25 base pairs and are the most abundant free‐circulating molecules in blood, although they are also present in urine, saliva, and CSF.^130^ They are released by tumors and host cells into the blood by apoptosis, necrosis, or active secretion through EVs or bound to plasma proteins.^131^ They control post‐transcriptional gene expression in a range of pathological and non‐pathological processes, including apoptosis, proliferation, differentiation, migration, and invasion, and are therefore a promising biomarker for cancer diagnosis.^132^ Particularly, miR‐21 and miR‐128 have been shown to be overexpressed in GBM patients, promoting tumor cell survival and invasiveness.^133^, ^134^ Furthermore, miR‐21 has been implicated in resistance to treatment, both against chemotherapy and radiotherapy.^135^

Despite its relevance, liquid biopsy based on the detection of circulating nucleic acids has been limited by their low amount in blood, as well as by current study techniques, in which there is no standardized protocol for sample extraction and purification.^136^ In this context, microfluidic techniques can be a valuable technique to isolate and analyze mostly ctDNA and avoid degradation or lysis of the molecules. For example, Kim et al. developed a lab‐on‐a‐disc system to isolate cfDNA, with which they were able to electromagnetically isolate 3 mL of blood in 30 min, decreasing the risk of sample degradation.^137^ Nonetheless, the concentration of cfDNA has wide variability based on patients, which becomes a limitation when only ctDNA is present in a small proportion. Gwak et al. developed a microfluidic vortex coupled to a gradient magnetic‐activated cfDNA sorter. With it, continuous perfusion of samples in the system solves the quantity limitation.^138^ Ou et al. created a system that combines microfluidic droplet portioning, fluorescent multiplex PCR, and their 3D large‐volume droplet counting technology to form a novel liquid biopsy system. It allows the analysis of total tumor DNA obtained from blood samples with 100% specificity.^139^ Moreover, some commercial tests have already been approved by the FDA for use in solid tumors. This is the case of Guardant360®, used to analyze ctDNA in blood^140^; FoundationOne® Liquid, which can detect ctDNA and ctRNA from blood samples, being able to analyze up to 300 genes^141^; and others such as Cobas EGFR Mutation test^142^ and Epi proColon®, specific for colorectal cancer.^143^ A study by Bruch et al. created a microfluidic biosensor to detect microRNAs (miR‐19b) in serum samples from children suffering from medulloblastoma, an aggressive brain tumor.^144^ They combined the CRISPR/Cas13a technology with a microfluidic sensor and electrochemical readout to detect miRNAs with high sensitivity and selectivity. They obtained a detection limit of 10 pM with less than 1 μL of sample, without nucleic acid amplification. However, there is currently no GBM‐specific system that has been approved for the identification of ctDNA or ctRNA. The improvement of microfluidic systems may be an important advance in the detection of these molecules, and the combination of circulating nucleic acids with on‐chip systems could open new avenues to molecularly isolate and analyze these molecules in GBM.

### 4.4 Tumor‐educated platelets in microfluidic models

Blood platelets play a critical role as local and systemic responders during tumorigenesis and metastasis.^145^, ^146^ When exposed to tumor cells, platelets undergo tumor‐induced education, leading to altered platelet behavior. This process, known as tumor‐educated platelet formation, results in platelets acquiring tumor‐specific molecular signatures, including proteins and RNA, which reflect the characteristics of the tumor.^147^, ^148^, ^149^ Due to their ability to capture key biological information from the tumor, TEPs emerge as a promising non‐invasive biomarker for liquid biopsy, offering valuable insights into cancer diagnosis and progression.

In 2011, Nilsson et al. identified EGFRvIII as a cancer‐associated RNA biomarker in gliomas.^150^ Later, Marx et al. observed increased CD63 expression and P‐selectin expression on circulating platelets in GBM patients.^151^ Best et al. performed RNA sequencing to identify differentially spliced RNA profiles in platelets from various patients and were able to predict healthy individuals and GBM patients (among other cancer types) with 96% accuracy, also identifying the location of the primary tumor with 71% accuracy.^152^ Additionally, they established that 100–500 pg. of total platelet RNA was sufficient for TEP‐based diagnostics. Sol et al. analyzed TEP‐spliced RNA profiles and confirmed that samples from GBM patients contain a distinct TEP profile compared to those with brain metastases. Moreover, they found that the GBM TEP fingerprint seems to decrease after surgical resection but reappears during tumor progression, indicating that it could be a useful method to distinguish true progression from pseudo‐progression.^145^

Regarding microfluidic devices, no devices focused on the detection of TEPs in GBM currently exist. Concerning other cancers, Hao et al. developed a microengineered microfluidic system in 2021 that allowed for stable perfusion of human platelets. This system created a model to predict platelet responsiveness before or during chemotherapy with doxorubicin.^153^ Li et al. described a chromatograph‐like microfluidic device that correlates platelet activation status with tumor progression, exhibiting sensitive, predictive potential for thrombotic events in cancer patients, thereby guiding well‐timed antithrombosis treatment.^154^ On the other hand, Ghosh et al. developed a device called the aTME‐Chip in 2024 to study the function of TEPs within the ovarian tumor microenvironment.^155^ Using this device, they observed that TEPs accelerated tumor angiogenesis. Furthermore, the effluents from the device allowed for the analysis of cytokine expression changes driven by platelet mechanisms.

### 4.5 Volatile organic compounds in microfluidic models

In addition to the aforementioned molecules, there is a group of metabolites potentially usable as liquid biomarkers, which are called VOCs. As described by the WHO,^156^ they are organic chemicals having an initial boiling point less than or equal to 250°C measured at a standard atmospheric pressure of 101.3 kPa.^157^, ^158^ VOCs have the ability to cross the BBB, making them ideal candidates for investigating alterations within the brain via blood biopsies.^33^ The entire set of VOCs generated by an organism is called “volatilome” and its study is known as “volatolomics.”^159^ These molecules are produced through cellular metabolism and are released into the bloodstream, where they can be detected directly, or through the lungs, urine, or skin.^160^, ^161^

VOCs are classified into five chemical functional groups: aldehydes, ketones, alcohols, hydrocarbons, and aromatic compounds.^159^, ^162^ They provide insights into biochemical processes triggered by oxidative stress, inflammation, apoptosis, or necrosis. VOCs linked to disease may result from the cascade of reactions that occur as the body responds to damage. This can either produce new metabolites or alter the levels of existing ones. For instance, the conversion of a normal cell into a cancerous cell is connected to distinct metabolic changes, such as the Warburg effect.^163^, ^164^, ^165^, ^166^, ^167^ In recent years, tumor‐specific VOCs are increasingly being introduced as tumor markers, and even standardized protocols for the collection and analysis have already been developed.^159^, ^160^, ^161^, ^168^, ^169^, ^170^, ^171^

Within the analytical methods that have been described for the detection of VOCs, solid phase microextraction gas chromatography mass spectrometry (SPME‐GC–MS) system is preferred to facilitate the identification and quantification of these compounds. However, other methods not based on mass spectrometry are being developed in parallel, such as electronic nose devices (enose),^172^, ^173^ bio‐sniffer^174^ and asymmetric ion mobility spectrometry.^160^, ^175^ Electronic nose devices are not sufficiently sensitive to detect those breath VOCs in the low ppb range, can be affected by ambient conditions (e.g., humidity and temperature), and have a limited lifetime.^172^ Ion mobility spectrometry presents an interesting compromise between classic analytical techniques, although it is still in an early developmental stage and has not been adopted widely within clinical trials.^160^

A number of articles have been published on the study of VOCs; most focused on breath analysis for the detection of lung cancer^160^, ^168^, ^172^, ^176^, ^177^, ^178^, ^179^, ^180^ or other types of cancer such as colorectal, breast, or prostate cancer.^170^, ^171^ However, VOCs emitted by breath are complex mixtures of elements influenced by external pollutants, which represent a significant loss of information. In contrast, the detection of VOCs in blood samples is a more selective method to exclude molecules from external sources. So far, there is not much information on the characterization of VOCs in blood from GBM patients. However, some studies have shown the presence of these metabolites in GBM patients with unfavorable outcomes.^181^ These VOCs were related to the pentose phosphate and Warburg effect pathways. Furthermore, the lipid profile of patients who experienced unfavorable outcomes revealed a higher heterogeneity in the abundance of lipids. Another study by Baranovičová et al. studied changes in basal blood plasma metabolites in patients with a primary GBM tumor, as well as their correlation with tumor grade. They found a significant increase in glycolytic metabolites such as glucose and pyruvate, and the levels of some metabolites such as tyrosine and phenylalanine were correlated exclusively with higher tumor grade, also known as glioblastoma.^182^

**FIGURE 3:**
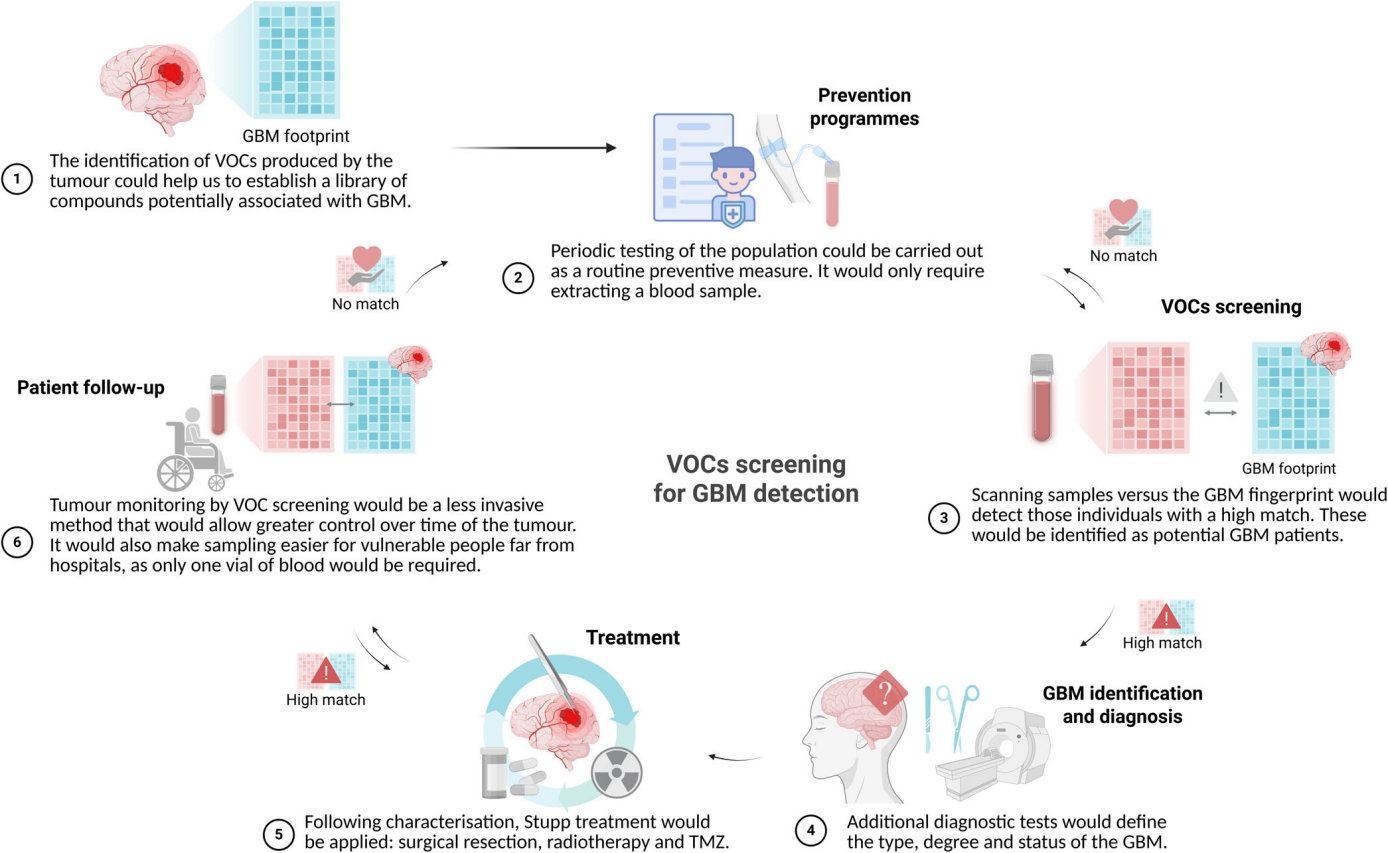
Schematic protocol of the potential future use of VOCs as a liquid biomarker in glioblastoma.

In terms of microfluidic approaches for glioblastoma, there are no studies to date using a microfluidic device for the direct detection of VOCs in blood or serum samples of GBM. The only study by Bayona et al. developed an organ‐on‐chip microfluidic technology for the recreation of the GBM tumor microenvironment and the detection and analysis of generated VOCs. This approach combined microfluidic technology with an SPME‐GC–MS system. In doing so, they were able to see significant increases in some aldehydes, phenols, and nitrogenous compounds, which correlated with those observed in patients.^76^ A recent study, although not focused on GBM, has developed a microfluidic device that can discriminate and classify six distinct types of gaseous aldehydes in the 100 parts per trillion range with 81% efficiency.^183^ This device integrates a concentrator platform together with reliable surface‐enhanced Raman scattering detection and could potentially be applied to the study of VOCs in GBM patients. Therefore, the detection of VOCs with the use of microfluidics could be of great relevance in the diagnosis and monitoring of tumors at an early stage (Figure 3).

## 5 CONCLUSIONS AND FUTURE PERSPECTIVES

Early diagnosis of brain tumors, particularly glioblastoma, remains a significant challenge due to the inaccessibility of the tumor and the nonspecific nature of its symptoms, which often overlap with more benign conditions. As a result, GBM is typically diagnosed at an advanced stage, limiting treatment options and worsening prognosis. There is a need to develop diagnostic methods that are less invasive, and above all, that allow frequent monitoring of patients or even screening at‐risk populations for early detection. Liquid biopsy has emerged as a promising method for GBM diagnosis, as it only requires a blood sample and well‐characterized GBM biomarkers to be implemented. While promising, detecting relevant biomarkers in the blood, such as CTCs, EVs, cfNAss, or VOCs, remains a challenge due to their low concentrations and the limitations of current isolation techniques. In this regard, microfluidic technologies, with their capacity for high‐precision fluid manipulation at the micro‐nano scale, offer a promising solution by improving the efficiency, selectivity, and sensitivity of biomarker detection in liquid biopsies. The ability to precisely control small liquid volumes enhances diagnostic accuracy and provides opportunities for real‐time monitoring of disease progression, which could ultimately contribute to better patient management.

As discussed in this review, several devices have already been developed for glioblastoma detection, primarily targeting CTCs and EVs. However, this still remains an underexplored field, and other blood‐based molecules could play a crucial role in improving the detection of GBM. Despite recent advances, the low abundance of biomarkers and limited sensitivity of current platforms present major obstacles. Improving these parameters is critical for the reliability of liquid biopsy tests, especially for detecting low‐abundance biomarkers. Furthermore, the development of standardized protocols for biomarker isolation, platform fabrication, and data analysis is crucial to ensure the consistency and reproducibility of microfluidic technologies across different laboratories and clinical settings. The absence of such protocols remains a significant barrier to the broader adoption of these technologies. Additionally, the costs associated with the development and commercialization of microfluidic devices, especially those that integrate advanced capabilities such as real‐time monitoring and multiplexing, are high. Optimizing these systems to be cost‐effective without compromising performance will be key for their widespread clinical adoption.

To overcome these challenges, interdisciplinary innovations are necessary. The combination of microfluidic‐based liquid biopsy with other emerging technologies, such as next‐generation sequencing, advanced imaging, and multi‐omics approaches, could provide a more comprehensive picture of GBM, facilitating earlier detection and more personalized treatment. Lab‐on‐a‐chip systems show particular promise for POC applications, enabling portable devices that facilitate real‐time, non‐invasive monitoring of GBM. This approach could make frequent patient assessments possible without the need for invasive procedures. The integration of droplet‐based microfluidics could further increase detection sensitivity by improving biomarker isolation and enabling high‐throughput analysis. Additionally, artificial intelligence could revolutionize data analysis, enabling more accurate interpretation of complex datasets and improving diagnostic performance. In fact, machine learning has already been used to improve CTC assessment^184^ and early cancer detection.^185^

As microfluidic platforms become more sensitive and refined, they may improve early detection rates and enable more personalized diagnostic approaches, leading to better‐targeted therapies. Moreover, the development of portable, POC devices could make real‐time, non‐invasive monitoring a feasible option for GBM patients. Together, the combination of microfluidics and liquid biopsies holds great promise for improving early diagnosis, patient management, and overall outcomes for patients suffering from glioblastoma.

## AUTHOR CONTRIBUTIONS

CB did the literature search, interpretation, and drafting of the manuscript. TR and COR revised the manuscript. IO revised the manuscript and supported the design of the paper. All authors read and approved the final manuscript.

## FUNDING INFORMATION

This work was supported by the Spanish Ministry of Economy and Competitiveness (MINECO fellowship, DIN 2020–011544); Ministry of Science and Innovation, the Agency, and the European Regional Development Fund (Project PID2021‐126051OB‐C41 funded by MCIN /AEI /10.13039/501100011033 / FEDER, UE). C.B. would like to thank Gobierno de Aragón (DGA) for the predoctoral funding. C.O.R. would like to acknowledge the financial support received from the Spanish Government through a research grant provided by the MINECO fellowship (DIN 2020–011544). The authors thank Gobierno de Aragón and Fondo Social Europeo for the financial help given to the TME lab group T62_23R.

## CONFLICT OF INTEREST STATEMENT

I. Ochoa is a promoter and consultant for BeOnChip S.L.

## Data Availability

Data sharing is not applicable to this article as no new data were created or analyzed in this study.
